# The Effect of Bariatric Surgery on Microvascular Structure and Function, Peripheral Pressure Waveform and General Cardiovascular Risk: A Longitudinal Study

**DOI:** 10.3390/jcm12237379

**Published:** 2023-11-28

**Authors:** Said Karimzad, Hala Shokr, Srikanth Bellary, Rishi Singhal, Doina Gherghel

**Affiliations:** 1 Optometry and Vision Sciences Research Group, Aston University, Birmingham B4 7ET, UK; saidekarimzad@hotmail.com (S.K.); hala.shokr@manchester.ac.uk (H.S.); 2 Pharmacy Division, Faculty of Biology, Medicine and Health, University of Manchester, Manchester M13 9PT, UK; 3 Aston Research Centre for Healthy Ageing (ARCHA), Aston University, Birmingham B4 7ET, UK; s.bellary@aston.ac.uk; 4 University Hospitals Birmingham NHS Foundation Trust, Birmingham B15 2GW, UK; rishi.singhal2@uhb.nhs.uk; 5 Division of Cardiovascular Sciences, University of Manchester, Manchester M13 9PL, UK

**Keywords:** bariatric surgery, microvascular function, retinal vessels calibre

## Abstract

Purpose: This study aims to assess the effect of bariatric surgery on retinal microvascular calibre, peripheral microvascular function, peripheral pressure waveforms, and the general cardiovascular disease (CVD) risk in obese individuals after undergoing Roux-en-Y gastric bypass (RYGB) surgery. Methods: A total of 29 obese participants were included in the study. All of the measurements were conducted at two time points: before and one year following the bariatric surgery procedure. General anthropometric data, as well as blood markers for glucose, cholesterol, and triglycerides were assessed in all individuals. In all participants, the Framingham risk score (FRS), and retinal vessel calibre measurements, using a Zeiss fundus camera and VesselMap software (ImedosSystems, Jena, Germany), were performed. Systemic arterial stiffness was measured by pulse wave analysis (PWA), and peripheral microvascular reactivity by way of digital thermal monitoring (DTM) in all participants. Results: As expected, various general anthropometric parameters, including body mass index (BMI), waist circumference and neck circumference, were significantly decreased post-surgery comparing to baseline in all individuals (all *p* < 0.001). In addition, their general CVD risk, as measured using FRS, was significantly improved (*p* < 0.001). At the retinal vascular level, central retinal artery equivalent (CRAE) as well as, central retinal vein equivalent (CRVE) had increased after surgery comparing to the baseline values (*p* = 0.003 and *p* = 0.007, respectively). In addition, both systemic arterial stiffness and peripheral microvascular reactivity had improved in all participants (*p* < 0.001 and *p* = 0.008 respectively). Conclusions: Our findings suggest that bariatric surgery has a positive effect on the overall vascular health, as well as on the general CVD risk of the obese patients undergoing this procedure.

## 1. Introduction

It is well known that, in general, obesity increases significantly the risk for cardiovascular disease (CVD) [1]. Excess adipose tissue induces several pathological changes, including dyslipidemia, elevated blood pressure (BP), oxidative stress, and increased inflammation, all very important risk factors for macro- and micro-vascular endothelial dysfunctions (ED), increased arterial stiffness, and atherosclerosis [2,3,4,5]. 

Both observational and interventional studies have demonstrated that loss of weight through various methods has positive effects on the patients’ cardiovascular health [6]. 

Among these various methods of weight-loss bariatric surgery is, to date, the most effective treatment proven to reduce not only the weight [7,8] but also the risk for CVD in patients that underwent this type of procedure [9,10]. It has been shown that reduction of certain metabolic irregularities begins as soon as few weeks or months after bariatric surgery, and even before a significant weight loss is achieved [11]. Nevertheless, and in contrast to the metabolic changes, the positive impact of this procedure on cardiovascular morbidity and mortality requires several years to become clinically apparent [12,13]. However, it is believed, that changes in several vascular and circulatory parameters, that point towards a possible reduced CVD risk, could be measurable much earlier post-surgery. To prove this assumption, various methods including assessments of macro- and microcirculation, inflammatory markers, lipid profiles and oxidative stress, were used [14,15,16,17]. But the question remains: what represents the most realistic measure of a high CVD risk? Today, the gold standard for absolute CVD risk is based on the FRS [18]. Other risk scores, such as the Prospective Cardiovascular Mὒnster (PROCAM) and the European Society of Cardiology Systematic Coronary Risk Evaluation scores (SCORE-2 and SCORE-OP) are also being used for the same purpose [19,20]. These scores, however, are based on “probabilistic calculations” derived from population-based studies and, as such, are thought to apply poorly at individual level [21]. For a more individual precision, the assessment of changes in easily accessible microcirculatory beds, such as the retinal vessels, have been tried [3,22]. Indeed, due to their anatomical and physiological similarities to those supplying the heart and the brain, the retinal vessels, have long emerged as strong candidates for CVD risk stratifications. The assessment of retinal arterioles’ diameters, measured as the central retinal arteriolar equivalent (CRAE) and central retinal venular equivalent (CRVE), are used routinely when assessing the degree of atherosclerosis [23], as well the presence of coronary heart disease (CAD) [24], the risk for stroke [25], or as indicators for cardiovascular mortality [26]. In addition, improvements of retinal vascular diameter have also been shown to be possible with positive health measures, including weight loss, suggesting a certain plasticity of the human microvasculature if treatments are applied early in the course of the disease [27]. In addition to the structure, the assessment of retinal vascular function also brings the benefit of providing an integrated and dynamic analysis that could be seen as specific for each individual and, as such, could be used for profiling a so-called *individualized vascular risk* for CVD.

There are many vascular markers that can be used for the patients’ categorization according to their specific CVD risk profiles. Due to their availability, however, reports on the hemodynamic alterations after bariatric surgery are variable and, to date, only few studies have looked in parallel at changes reflected in multiple vascular beds after this procedure [17,28]. Therefore, for a more complete picture, the objective of this study was to evaluate the impact of weight loss on retinal microvascular calibre, peripheral microvascular function, peripheral pressure waveforms, and the overall risk of CVD in a group of obese individuals undergoing Roux-en-Y gastric bypass (RYGB) surgery.

## 2. Materials and Methods

### 2.1. Patient Recruitment

This longitudinal study included 40 patients recruited from the University Hospitals Birmingham NHS Foundation Trust’s Weight Management Clinic.

Prior to the study ethical approval was received from Coventry & Warwickshire West Midlands research ethics committee, Heart of England Foundations trust (HEFT) NHS Research Ethics Committees, University Hospitals Birmingham as well as the Aston University Life and Health Sciences Ethics Committee (protocol code 932, Aston University, UK) and all procedures were designed and conducted in accordance with the tenets of the Declaration of Helsinki.

Successive diagnosed obese patients with BMI > 40 kg/m^2^ were recruited from the weight management clinics by a specialist consultant and written informed consent was received from all subjects before entry into the study. 

The inclusion criteria for the participants was individuals classified as morbidly obese as per the international guidelines on obesity with a BMI value of greater than 40 kg/m^2^, scheduled for bariatric surgery [29]. To reduce any treatment bias, only obese participants that have never received any weight reduction treatment were enrolled. 

Study exclusion criteria were defined as a positive diagnosis of CVD including CAD, peripheral artery disease (PAD), and aortic atherosclerosis. Similarly, individuals with cerebrovascular disease, diabetes, or other metabolic disorders such as dyslipidemia were excluded from the study. Lifestyle and non-pharmacologically controlled systemic hypertension (below 140/90 mmHg according to the European Society of Cardiology guidelines [30] were neither an inclusion nor exclusion criteria. However, individuals using any vasoactive medications such as dietary supplements containing vitamins or antioxidants and bronchodilators were also excluded from the study. 

Potential participants were also screened for ocular diseases and were excluded from the study if they had a refractive error of more than ±3 DS and more than ±1 DC equivalent, intra-ocular pressure (IOP) greater than 21 mmHg, cataract, or any other media opacities, as well as history of intra-ocular surgery or any form of retinal or neuro-ophthalmic disease affecting the ocular vascular system and could interfere with the study’s measurements. Individuals with sings of hypertensive retinopathy at the initial fundus examination were also excluded.

All included participants underwent RYGB surgery according to a standard technique. Baseline measurements were performed 1 month prior to the participant undergoing surgery and the follow-up was performed 12 months after the procedure. The assessments are detailed below.

### 2.2. Demographic and General Health History 

Participants who met the inclusion criteria and had provided informed consent were requested to complete a demographic and general health history questionnaire detailing their age, gender, ethnicity, personal and family history of illness, medication, daily diet, tobacco and alcohol consumption, and physical activity routine.

Anthropometric measures including height and weight were recorded using standard procedures. Body mass index (BMI), waist, and neck circumference (WC, NC, respectively) were also assessed. Systolic blood pressure (SBP), diastolic blood pressure (DBP) and heart rate (HR) were measured using an automatic blood pressure (BP) monitor (UA-767; A&D Instruments Ltd., Abingdon, UK) to determine mean arterial pressure (MAP = 2/3 DBP + 1/3 SBP) the European Society of Cardiology guidelines [31]. Intraocular pressure (IOP) in mmHg readings were obtained using non-contact tonometry (Pulsair; Keeler Ltd., Windsor, UK).

**Figure 1:**
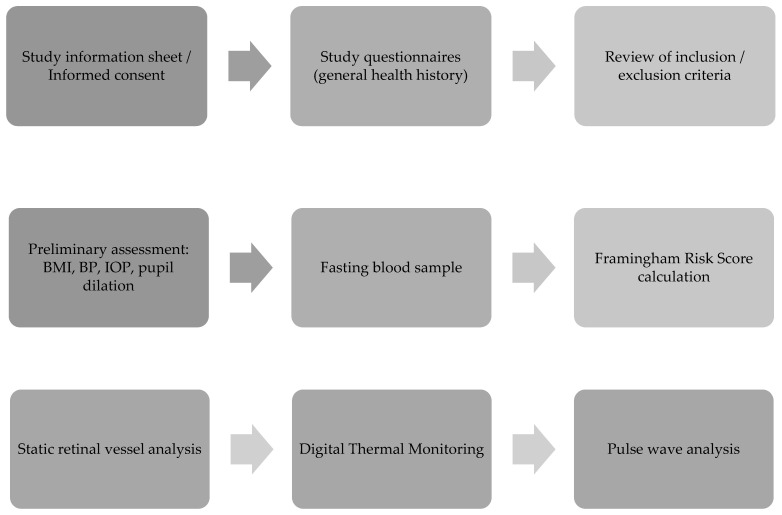
Patient visit protocol; BMI, body mass index; BP, blood pressure; IOP, intraocular pressure.

All study-related assessments were performed between 8 and 11 am following an overnight fast for at least 10 to 12 h, which included refraining from alcohol and caffeine. Study procedures were performed as outlined in the flowchart (Figure 1) with details on techniques, procedures, and data analysis provided in the following sections. 

### 2.3. Blood Analyses

Blood and plasma samples drawn from the antecubital fossa vein were assessed for fasting glucose (GLUC), triglycerides (TG), total cholesterol (T-CHOL) and high-density lipoprotein cholesterol (HDL-C), using the Reflotron Desktop Analyzer (Roche Diagnostics, Welwyn Garden City, UK), where test strips designed for the specific determination of clinical chemistry parameters using undiluted specimen material are used. Low-density lipoprotein cholesterol (LDL-C) values were calculated as per the Friedewald equation [32]. 

### 2.4. Framingham Risk Score (FRS) 

The FRS for each individual was calculated according to already published formula [33]. All screened individuals were classified, using the absolute CVD risk percentage over 10 years, as low risk (<10%), intermediate risk (10–20%) and high risk (>20%) [34]. 

### 2.5. Quantification of Retinal Vessel Calibre (CRAE, CRVE and AVR)

**Figure 2:**
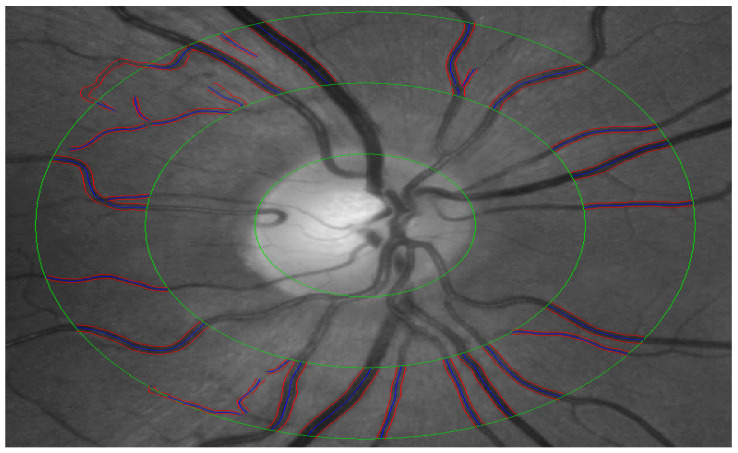
An example of the fundus photograph used for retinal vessels grading.

Monochromatic retinal images with the optic nerve head centered were obtained using a Zeiss FF450+ fundus camera. CRAE, CRVE and AVR were calculated semi-automatically using the software VesselMap (ImedosSystems, Jena, Germany) according to an already published method [35]. In brief, following the image selection, a ring is placed around the ONH with 2 further concentric rings with each ½ DD and 1 DD distant from the ONH ring around it (Figure 2). After that, few largest retinal arteries and veins passing through the outer ring segment are selected for the analysis [35].

### 2.6. Digital Thermal Monitoring (DTM)

Peripheral microvascular reactivity at the level of the fingertips was assessed using VENDYS 5000 BCE DTM system (Endothelix, Inc., Houston, TX, USA). The test is conducted with the patient at rest for 30 min in the supine position, in a quiet, dimmed room with ambient temperature of 22 °C to 26 °C. VENDYS DTM probes are affixed to the index finger of each hand and after a period of stabilization of basal skin temperature (defined as stabilization within a 0.05 °C threshold) the temperature is measured in the index fingers of both hands (of which the right arm only is subjected to occlusion-hyperaemia) with an automated, operator-independent protocol. The right upper arm cuff is rapidly inflated to ≥50 mmHg above systolic pressure for 5 min and then rapidly deflated to invoke reactive hyperaemia distally. Thermal tracings are measured continuously and digitized automatically using a computer-based thermometry system with 0.006 °C thermal resolution. 

**Figure 3:**
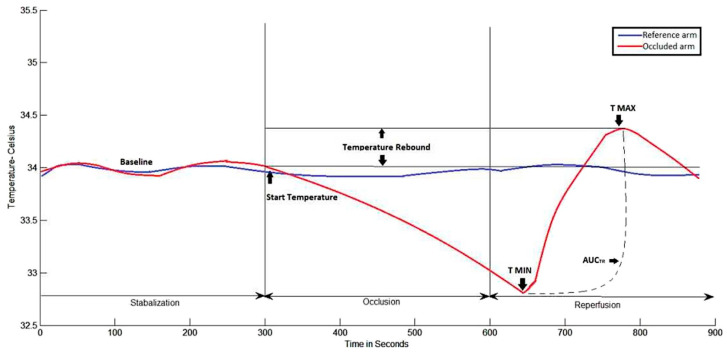
Graphical representation of the Digital Thermal Monitor software analysis. Abbreviations: T MAX, maximum temperature; TMIN, minimum temperature; AUCTR, Area under the curve temperature rebound.

Dual channel temperature data simultaneously acquired at a 1 Hz sample rate (Figure 3) show a representative example of a temperature-time trace and the primary DTM-derived measures, which is related to thermal debt and recovery that were recorded and calculated. Temperature rebound (TR): maximum temperature-start temperature (just before cuff inflation); adjusted temperature rebound (aTR): temperature rebound/start temperature; area under the curve temperature rebound (AUCTR): area under the curve between maximum temperature and minimum temperature. The post-occlusive adjusted temperature rebound aTR determined by the software algorithm is directly associated with the extent of the subject’s vascular reactivity. An aTR below 1 is considered poor cardiovascular reactivity, whereas an aTR between 1 and 2 is considered intermediate vascular reactivity and an aTR of >2 is considered healthy vascular reactivity [36].

### 2.7. Pulse Wave Analysis (PWA)

PWA was conducted in accordance with an established protocol using the validated SphygmoCor device (AtCor Medical/PWV Medical Pty Ltd., Sydney, Australia) [37]. The patient’s radial pulse was first located just below the wrist creases at the base of the thumb and the SphygmoCor transducer or high-fidelity pressure sensor was flattened over this site with slight pressure to generate a signal representative of the intravascular pulse in the radial artery. Reasonable confidence in readings was gained when pressure waves were consistent from beat to beat and with characteristics to be expected in the artery (sharp upstroke to the first systolic peak, sharp cleft, and near-exponential pressure decay in late diastole). The pulsatile radial waveform was then calibrated against SBP and DBP readings by the in-built software, and mathematically transformed using a transfer function to reconstruct the aortic waveform from which a range of central cardiovascular parameters can be derived.

### 2.8. Statistical Analysis

All analyses were conducted using Statistica^®^ software (StatSoft Inc., Version 16, Tulsa, OK, USA). We assessed the distribution of continuous variables with the Shapiro-Wilk test. In cases where normality could not be confirmed, we applied appropriate data transformations or employed non-parametric statistical methods. For univariate associations, Pearson’s correlation was used for normally distributed data and Spearman’s correlation for non-normally distributed data. The Bonferroni correction was utilized to control the familywise error rate. Furthermore, forward stepwise regression analyses were performed to evaluate the impact of clinical parameters and circulating markers, such as CHOL, HDL, LDL, and HbA1c, on the measured vascular reactivity variables. These factors were also considered as statistical confounders in the multivariate analysis to ensure the precision and reliability of the findings, given their potential influence on CVDs. Differences between groups in clinical characteristics, retinal caliber, and systemic vascular reactivity measures were assessed using *t*-tests or analysis of covariance (ANCOVA) where applicable. Statistical significance was defined as *p* < 0.05.

### 2.9. Power Calculations

The sample size was calculated using the software G power (University of Kiel, version 3.1.6, Kiel, Germany). 

Based on previous studies, retinal vessel calibres in healthy individuals have a mean diameter of 202.3 μm that vary 10–15 μm but can change up to 30% with pathology [9,38,39,40] therefore a similar difference between groups was expected in this study. Previous studies also report a pulse wave analysis change by 4.8% for every 10 bpm in heart rate [41]. Additionally, DTM measurements in healthy individuals have been known to change in temperature between 2.4 ± 1.60 degrees [42]. Hence, it was anticipated that a paired *t*-test or ANCOVA would be required in this study and given the uniqueness of the comparisons being made with regards to retinal and systemic vascular parameters, sample size calculations were based on a number of assumptions. Based on Cohen’s standardized classification of effect sizes: small effect = 0.10; medium effect = 0.25; large effect = 0.40 it was expected that a large size effect of at least 0.40 would be observed, and in order to provide a statistical power of 95% with the number of study groups specified as 2 and an alpha-level set at 0.05, a sample size of n = 23 was recommended.

## 3. Results

### 3.1. Clinical Characteristics

A total of 40 participants were initially screened for study inclusion and had completed all of the baseline measurements. Eleven participants were, however, lost to follow-up. The remaining 29 participants were included in the final analysis.

The clinical characteristics of the study groups are summarized in Table 1. Bariatric surgery led to statistically significant decreases in mean BMI, along with reductions in WC and NC. Furthermore, we noted significant reductions in SBP, DBP, HR, and similarly, IOP also exhibited a significant reduction within our cohort. Bariatric surgery also resulted in significant improvements in mean fasting blood glucose level GLUC. In addition, lipid profile also showed improvements with reduction in total cholesterol, LDL-C, TG and increases in HDL-C. An amelioration of CVD risk was also observed with a 42% reduction in FRS. 

### 3.2. Retinal Microcirculation Assessment 

After the 12-month post-surgery period, there was a significant increase in patients’ mean CRAE and CRVE, as shown in Table 2. This resulted in a decrease in the AVR; however, this change did not attain statistical significance.

### 3.3. Assessments of Peripheral Vascular Function and Peripheral Pressure Waveforms

Table 3 provides a summary of the systemic investigations conducted using DTM and PWA at both baseline and follow up. DTM parameters, notably aTR and AUCtr were significantly improved after the surgery, whereas AIx showed a reduction following the procedures.

### 3.4. Correlations between Retinal Vessel Calibres and Systemic Parameters

**Figure 4:**
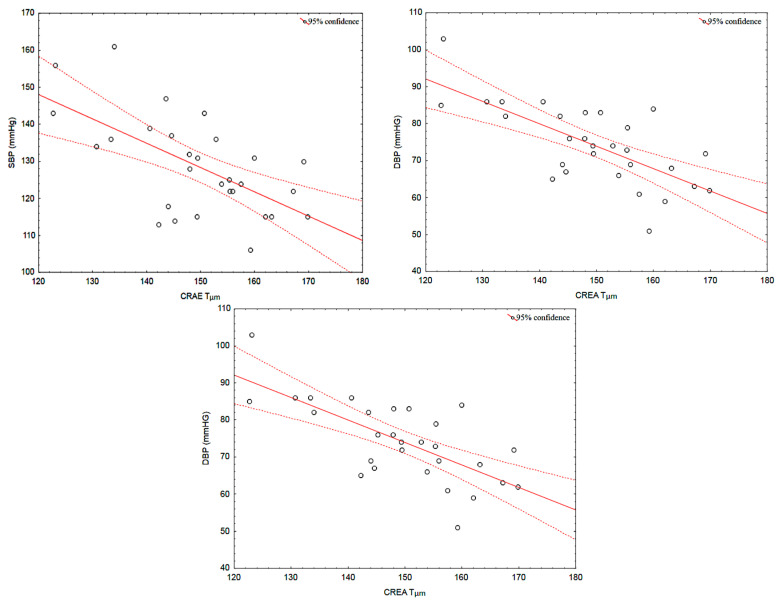
Correlation between SBP, DBP, HDL-C, and CRAE in the follow up subjects. Abbreviations: SBP, systolic blood pressure; DBP, diastolic blood pressure; HDL-C, high density lipoprotein cholesterol; CRAE, central retinal artery equivalent.

After surgery, the observed improvements in SBP and DBP correlated with those recorded in CRAE (r = −0.62, *p* < 0.001, r = −0.70, *p* < 0.001). Moreover, the observed in HDL-C were also associated with the increases in CRAE (r = 0.46, *p* = 0.011 respectively) (Figure 4). These correlations were not present at baseline (all *p* > 0.05). No other correlations were identified.

## 4. Discussion

In this longitudinal study, we utilized a self-control design to examine the impact of substantial weight loss on various vascular beds and the overall CVD risk at twelve-month post bariatric surgery in a group of 29 obese individuals undergoing the RYGB procedure. In agreement with previous studies, our results show that, in addition to an overall positive effect on the general risk for CVDs, bariatric surgery had also showed an improvement of the retinal arteries diameter [43,44]. Indeed, narrowing of the retinal arteries represents a well-known marker for chronic damage due to elevated BP [45,46,47,48,49] and large observational studies have already established a strong association between abnormal AVR and risk for CVD [50,51]. As such, by showing an improvement in the retinal arteries diameter, we have demonstrated a positive effect of this procedure on the general CVD risk. In addition, the observed enhancement of the retinal arteries caliber following bariatric surgery could also suggest that abnormalities of the microvascular diameters are, potentially, reversible to the normal or near normal values when the procedure is administered early in the course of the disease. Similar conclusions were drawn by Viljanen et al. [27]. In 22 obese individuals undergoing laparoscopic Roux-en-Y gastric bypass or a sleeve gastrectomy, these researchers were able to demonstrate that, at 6-months post procedure, the patients showed an improvement in the retinal artery caliber. However, and contrary to our findings, the above-mentioned study has reported a decrease in the venular diameter, rather than an increase, as per our report. Wider venules are a marker of obesity [52] and are associated with an increase in risk for diabetes [53]. It is not clear why, in our sample, the venular diameter has increased post-procedure? More research is necessary to understand the implication of this observation.

Similar to previous research [16,44,54,55,56], our subjects have also shown a marked improvement in PWA parameters and, as such, a reduction of the general arterial stiffness (AS). Nevertheless, other studies did not find such changes in the AS. Indeed, in their study, Jamialahmadi et al., have observed a significant decline of PWV, post-bariatric surgery [16]. It is well-known that AS may be one of the mechanisms by which obesity increases cardiovascular risk independently of traditional risk factors [55]. However, this parameters can be assessed using various methodologies. In the light of the above variable results, however, we could conclude that the interpretation of arterial stiffness improvements should be made in the context of each study and their used method for assessing AS. 

It is well-known that both weight loss as such [57] as well as bariatric surgery [17,58] improve macrovascular endothelial function as assessed by flow mediated dilation (FMD) procedure. As an example, in a 12-months prospective study, looking at the dietary and/or surgical-induced weight loss on the vascular function, Bigornia et al. have shown that sustained weight loss has improved vascular function and metabolic parameters in severely obese patients [59]. The authors concluded that the observed reversal in macrovascular endothelial dysfunction could potentially indicate a reduction in these patients’ cardiovascular risk. In a more recent study, Gockce et al. found that that bariatric intervention results in improvements not only of the macro- but also of the microvascular function of obese individuals [28]. As the vascular function can show much earlier improvements that changes in the structure after weight loss, were have also looked to assess microcirculatory functionality in our cohort. As a result, and by using the DTM method, we have demonstrated that the fingertip aTR increased at 12 months after bariatric surgery, possibly though an increase in nitric oxide (NO) bioavailability. Indeed, reactive hyperaemia after a period of ischemia represents a physiologic response of the vasculature and endothelial system strongly dependent on the degree of NO release following local ischemic stress [60,61]. In addition, our patients also demonstrated an increase in AUCtr, which corresponds to faster temperature rebound response after induced ischemia, and, as such, represents a marker of improved endothelial function at the peripheral microvascular level [62]. Therefore, our results confirmed the positive effect of bariatric interventions on microvascular function, similarly to previous studies [28].

It is of special note that in our cohort, after bariatric surgery, the observed reduction in the systemic BP correlated with the changes observed in the retinal vascular caliber. Indeed, it is well-known the detrimental effect of high systemic BP on microcirculation, including the retinal microvessels [63]. However, to our knowledge, this is the first time when a reduction in BP post-bariatric surgery is reported to correlate with changes in the retinal microvascular caliber. This finding is of extreme importance and suggest that the improvements in micro- and macrocirculation after this procedure are, possibly, dynamic and interdependent. Moreover, we have also shown that an increase in HDL-C level correlated with the observed improvement in retinal vessels caliber. It is well-known that HDL-C contributes to a normal vascular endothelial function, stimulates prostacyclin production (which is both vasodilatory and antithrombotic), inhibits endothelial cell apoptosis, decreases platelet aggregability, inhibits LDL oxidation, and reduces inflammation [64,65]. As a result, low serum levels of HDL-C are commonly encountered in patients with coronary artery disease (CAD) [66]. Low levels of HDL-C are also seen in patients with high risk of retinal artery [67] or vein occlusions [68,69]. Our results are the first to show that an improvement in HDL-C levels after bariatric surgery is associated with positive changes in the retinal artery diameters, showing not only a possible general reduction in CVD risk in these patients [70]. 

It is important to acknowledge that this study could have suffered from several potential limitations. One of these limitations is the relatively small sample size. Additionally, the 12-month interval elapsed between the baseline and follow-up assessments was, seemingly, long. However, Habib et al., demonstrated that although changes in vascular parameters were observed as soon as six months after surgery, no further changes occurred thereafter [71] and, as such, we do not see the length of the time between the two assessments as a limitation for this study. However, a very important limitation could be that we have not accounted for changes in the diet and physical activity that our patients have possibly embraced post-surgery. Indeed, the effects of these changes could have had important effects on our measured parameters. Other studies, however, have also omitted these effects when studying the structural, functional, and circulatory effects of weight loss after bariatric surgery. Nevertheless, for a full picture, further studies to include all such variable are necessary. 

In conclusion, our study shows that bariatric surgery results in measurable hemodynamic improvements at multiple levels that could, collectively, result in a general reduction in CVD risk in obese individuals undergoing such procedure. 

## Figures and Tables

**Table 1:** Participants’ baseline and follow up clinical characteristics.

Variables	Baseline Mean (SD)	Follow Up Mean (SD) (12 Months after the RYGB Surgery)	*p*-Value
BMI (kg/m^2^)	49.2 (7.69)	38.38 (7.84)	<0.001 *
WC cm	137.17 (20.42)	112.04 (24.21)	<0.001 *
NC cm	42.58 (4.74)	38.03 (4.18)	<0.001 *
SBP (mmHg)	144.24 (14.35)	128.75 (13.23)	<0.001 *
DBP (mmHg)	78.96 (11.35)	73.34 (10.84)	0.039 *
MAP (mmHg)	100.72 (10.58)	92.48 (10.94)	<0.001 *
HR (bpm)	74.10 (13.45)	69.17 (9.88)	0.046 *
IOP (mmHg)	15 (2.40)	12.58 (1.95)	<0.001 *
OPP	52.14 (8.01)	49.06 (8.11)	0.026 *
CHOL (mmol/L)	4.90 (1.21)	4.53 (0.97)	0.003 *
HDL-C (mmol/L)	1.24 (0.35)	1.51 (0.40)	<0.001 *
LDL-C (mmol/L)	2.97 (1.01)	2.49 (0.81)	<0.001 *
TG (mmol/L)	1.44 (0.72)	1.13 (0.50)	0.002 *
GLUC (mmol/L)	5.75 (0.75)	5.37 (0.42)	0.004 *
FRS%	12.00 (7.73)	6.51 (5.17)	<0.001 *

Abbreviations: SD, standard deviation; BMI, body mass index; WC, waist circumference; NC, neck circumference; SBP, systolic blood pressure; DBP, diastolic blood pressure; MAP, mean arterial pressure; HR, heart rate; IOP, intraocular pressure; OPP, ocular perfusion pressure; CHOL, total cholesterol; HDL-C, high density lipoprotein cholesterol; LDL-C, low density lipoprotein cholesterol; TG, triglycerides; GLUC, glucose; FRS, framingham risk score. * Significant *p*-values where *p* < 0.05 was considered significant.

**Table 2:** Summary of retinal vessel calibres before and after the procedure.

Parameter	Baseline Mean (SD)	Follow Up Mean (SD) (12 Months after the RYGB Surgery)	*p*-Value
CRAE (μm)	143.70 (13.97)	149.31 (12.05)	0.003 *
CRVE (μm)	204.72 (23.26)	213.24 (20.75)	0.007 *
AVR	0.67 (0.08)	0.65 (0.08)	0.068

Abbreviations: SD, standard deviation; CRAE, central retinal artery equivalent CRVE, central retinal vein equivalent; AVR, arteriolar to venular diameter ratio. * Significant *p*-values are indicated in bold where *p* < 0.05 was considered significant.

**Table 3:** Summary of Peripheral Vascular Function measurements before and after the procedure.

Parameter	Baseline Mean (SD)	Follow Up Mean (SD) (12 Months after the RYGB Surgery)	*p*-Value
aTR	1.71 (1.02)	2.39 (1.04)	0.008 *
AUCtr	264.51 (159.18)	360.96 (181.40)	0.025 *
Alx	25.79 (8.85)	20.10 (10.45)	<0.001 *

Abbreviations: SD, standard deviation; TR, temperature rebound; aTR, adjusted temperature rebound; AUCtr, area under the curve temperature rebound; Alx, augmentation index. * Significant *p*-values are indicated in bold where *p* < 0.05 was considered significant.

## Data Availability

The data presented in this study are available on request from the corresponding author. The data are not publicly available due to ethical considerations.
